# Supplementary material for: Multi‐dataset identification of innovative feature genes and molecular mechanisms in keratoconus
Source: J Cell Mol Med. 2024 Sep 19;28(18):e70079. doi: 10.1111/jcmm.70079 (PMC11412914; doi:10.1111/jcmm.70079)
Supplement: Supplementary file 2 — Material S2. [file JCMM-28-e70079-s002.zip › 7.GSEA analysis/3.DBNDD1/KEGG_ENDOCYTOSIS.html]

Details for gene set KEGG\_ENDOCYTOSIS[GSEA]

|  || Dataset | DBNDD1.group.cls#h\_versus\_l |
| Phenotype | group.cls#h\_versus\_l |
| Upregulated in class | l |
| GeneSet | KEGG\_ENDOCYTOSIS |
| Enrichment Score (ES) | -0.23886505 |
| Normalized Enrichment Score (NES) | -1.2665546 |
| Nominal p-value | 0.18609408 |
| FDR q-value | 0.6361566 |
| FWER p-Value | 0.974 |
Table: GSEA Results Summary

  

Fig 1: Enrichment plot: KEGG\_ENDOCYTOSIS      
 Profile of the Running ES Score & Positions of GeneSet Members on the Rank Ordered List

  

| SYMBOL | TITLE | RANK IN GENE LIST | RANK METRIC SCORE | RUNNING ES | CORE ENRICHMENT || 1 | PDGFRA | na | 3213 | 0.243 | -0.0750 | No |
| 2 | IL2RB | na | 3295 | 0.239 | -0.0595 | No |
| 3 | CXCR2 | na | 3338 | 0.238 | -0.0431 | No |
| 4 | CBLB | na | 4045 | 0.212 | -0.0477 | No |
| 5 | ACAP1 | na | 4355 | 0.202 | -0.0417 | No |
| 6 | AGAP2 | na | 4640 | 0.192 | -0.0357 | No |
| 7 | RAB11FIP4 | na | 4901 | 0.183 | -0.0296 | No |
| 8 | F2R | na | 5139 | 0.176 | -0.0234 | No |
| 9 | PSD2 | na | 5720 | 0.158 | -0.0285 | No |
| 10 | ARRB1 | na | 5893 | 0.153 | -0.0221 | No |
| 11 | HSPA2 | na | 5905 | 0.153 | -0.0110 | No |
| 12 | CLTC | na | 6408 | 0.139 | -0.0152 | No |
| 13 | ARAP1 | na | 6423 | 0.139 | -0.0053 | No |
| 14 | ASAP3 | na | 6521 | 0.136 | 0.0020 | No |
| 15 | HSPA8 | na | 7790 | 0.108 | -0.0267 | No |
| 16 | HSPA1L | na | 7968 | 0.104 | -0.0241 | No |
| 17 | EPS15 | na | 8105 | 0.102 | -0.0205 | No |
| 18 | IGF1R | na | 8164 | 0.100 | -0.0148 | No |
| 19 | HLA-F | na | 8547 | 0.094 | -0.0189 | No |
| 20 | EPN3 | na | 8656 | 0.092 | -0.0152 | No |
| 21 | PARD6G | na | 8803 | 0.089 | -0.0128 | No |
| 22 | ARAP2 | na | 9426 | 0.079 | -0.0249 | No |
| 23 | NEDD4L | na | 9725 | 0.075 | -0.0280 | No |
| 24 | PIP4K2B | na | 9747 | 0.075 | -0.0230 | No |
| 25 | MDM2 | na | 9756 | 0.075 | -0.0177 | No |
| 26 | NTRK1 | na | 10107 | 0.070 | -0.0226 | No |
| 27 | AP2A2 | na | 10114 | 0.070 | -0.0176 | No |
| 28 | RAB31 | na | 10123 | 0.070 | -0.0127 | No |
| 29 | IL2RG | na | 10291 | 0.067 | -0.0125 | No |
| 30 | SH3GL2 | na | 10439 | 0.065 | -0.0119 | No |
| 31 | PDCD6IP | na | 10897 | 0.059 | -0.0207 | No |
| 32 | MVB12B | na | 10910 | 0.059 | -0.0167 | No |
| 33 | PSD4 | na | 10968 | 0.059 | -0.0140 | No |
| 34 | ADRB2 | na | 11057 | 0.058 | -0.0123 | No |
| 35 | DAB2 | na | 11524 | 0.052 | -0.0219 | No |
| 36 | CHMP4A | na | 11613 | 0.051 | -0.0207 | No |
| 37 | FGFR4 | na | 11906 | 0.048 | -0.0256 | No |
| 38 | CHMP6 | na | 11945 | 0.048 | -0.0231 | No |
| 39 | RET | na | 11988 | 0.047 | -0.0208 | No |
| 40 | VPS37B | na | 12177 | 0.045 | -0.0229 | No |
| 41 | FGFR3 | na | 12198 | 0.045 | -0.0201 | No |
| 42 | CBL | na | 12212 | 0.045 | -0.0172 | No |
| 43 | PARD6A | na | 12250 | 0.045 | -0.0149 | No |
| 44 | CHMP4B | na | 12309 | 0.044 | -0.0133 | No |
| 45 | RAB22A | na | 12551 | 0.042 | -0.0171 | No |
| 46 | STAM2 | na | 12799 | 0.040 | -0.0213 | No |
| 47 | TFRC | na | 12909 | 0.039 | -0.0216 | No |
| 48 | ACAP2 | na | 12959 | 0.039 | -0.0201 | No |
| 49 | EPN1 | na | 13006 | 0.038 | -0.0186 | No |
| 50 | ERBB4 | na | 13020 | 0.038 | -0.0162 | No |
| 51 | HSPA1A | na | 13025 | 0.038 | -0.0135 | No |
| 52 | EEA1 | na | 13121 | 0.037 | -0.0135 | No |
| 53 | CHMP3 | na | 13159 | 0.037 | -0.0118 | No |
| 54 | EPN2 | na | 13751 | 0.032 | -0.0265 | No |
| 55 | GRK6 | na | 13765 | 0.032 | -0.0245 | No |
| 56 | RAB5C | na | 14015 | 0.030 | -0.0295 | No |
| 57 | PIKFYVE | na | 14064 | 0.030 | -0.0287 | No |
| 58 | PRKCZ | na | 14131 | 0.029 | -0.0285 | No |
| 59 | PSD3 | na | 14156 | 0.029 | -0.0270 | No |
| 60 | VPS25 | na | 14189 | 0.029 | -0.0258 | No |
| 61 | RUFY1 | na | 14202 | 0.028 | -0.0241 | No |
| 62 | CLTA | na | 14326 | 0.028 | -0.0256 | No |
| 63 | ACAP3 | na | 14571 | 0.026 | -0.0307 | No |
| 64 | VPS4A | na | 14577 | 0.026 | -0.0289 | No |
| 65 | CCR5 | na | 14673 | 0.025 | -0.0298 | No |
| 66 | PSD | na | 14954 | 0.023 | -0.0362 | No |
| 67 | ARFGAP1 | na | 15070 | 0.022 | -0.0379 | No |
| 68 | VPS36 | na | 15113 | 0.022 | -0.0375 | No |
| 69 | LDLRAP1 | na | 15167 | 0.022 | -0.0374 | No |
| 70 | ARAP3 | na | 15271 | 0.021 | -0.0388 | No |
| 71 | STAMBP | na | 15363 | 0.020 | -0.0400 | No |
| 72 | SMAP1 | na | 15414 | 0.020 | -0.0399 | No |
| 73 | PLD1 | na | 15421 | 0.020 | -0.0386 | No |
| 74 | CBLC | na | 15442 | 0.020 | -0.0378 | No |
| 75 | SNF8 | na | 15592 | 0.019 | -0.0407 | No |
| 76 | RAB4A | na | 15801 | 0.017 | -0.0454 | No |
| 77 | IQSEC2 | na | 15886 | 0.017 | -0.0466 | No |
| 78 | FLT1 | na | 15887 | 0.017 | -0.0454 | No |
| 79 | PIP5K1C | na | 16052 | 0.015 | -0.0490 | No |
| 80 | PARD3 | na | 16208 | 0.014 | -0.0524 | No |
| 81 | GIT1 | na | 16349 | 0.014 | -0.0555 | No |
| 82 | USP8 | na | 16508 | 0.013 | -0.0591 | No |
| 83 | ITCH | na | 16615 | 0.012 | -0.0613 | No |
| 84 | CHMP2B | na | 16687 | 0.011 | -0.0625 | No |
| 85 | AGAP1 | na | 16694 | 0.011 | -0.0618 | No |
| 86 | VPS28 | na | 16716 | 0.011 | -0.0616 | No |
| 87 | SH3GLB2 | na | 16733 | 0.011 | -0.0613 | No |
| 88 | TRAF6 | na | 17024 | 0.009 | -0.0690 | No |
| 89 | MVB12A | na | 17283 | 0.008 | -0.0759 | No |
| 90 | ARFGAP2 | na | 17395 | 0.007 | -0.0786 | No |
| 91 | HLA-E | na | 17418 | 0.007 | -0.0787 | No |
| 92 | RBSN | na | 17467 | 0.006 | -0.0796 | No |
| 93 | RAB11FIP3 | na | 17528 | 0.006 | -0.0809 | No |
| 94 | AP2M1 | na | 17638 | 0.005 | -0.0836 | No |
| 95 | VPS4B | na | 17697 | 0.005 | -0.0849 | No |
| 96 | VPS37C | na | 17953 | 0.003 | -0.0921 | No |
| 97 | LDLR | na | 18025 | 0.002 | -0.0940 | No |
| 98 | RAB11B | na | 18042 | 0.002 | -0.0943 | No |
| 99 | SH3GL1 | na | 18043 | 0.002 | -0.0941 | No |
| 100 | DNM2 | na | 18068 | 0.002 | -0.0946 | No |
| 101 | PRKCI | na | 18135 | 0.002 | -0.0964 | No |
| 102 | VTA1 | na | 18167 | 0.002 | -0.0972 | No |
| 103 | KDR | na | 18209 | 0.001 | -0.0983 | No |
| 104 | HSPA1B | na | 18247 | 0.001 | -0.0993 | No |
| 105 | TSG101 | na | 18307 | 0.001 | -0.1009 | No |
| 106 | RAB11FIP1 | na | 18548 | -0.001 | -0.1078 | No |
| 107 | CHMP5 | na | 18566 | -0.001 | -0.1082 | No |
| 108 | ARRB2 | na | 18679 | -0.002 | -0.1113 | No |
| 109 | HRAS | na | 18984 | -0.004 | -0.1197 | No |
| 110 | AP2S1 | na | 18992 | -0.004 | -0.1196 | No |
| 111 | EGF | na | 19333 | -0.007 | -0.1289 | No |
| 112 | RABEP1 | na | 19364 | -0.007 | -0.1292 | No |
| 113 | CHMP2A | na | 19447 | -0.008 | -0.1310 | No |
| 114 | ARF6 | na | 19486 | -0.008 | -0.1315 | No |
| 115 | RAB5A | na | 19553 | -0.009 | -0.1327 | No |
| 116 | KIT | na | 19563 | -0.009 | -0.1323 | No |
| 117 | HLA-G | na | 19797 | -0.011 | -0.1382 | No |
| 118 | HLA-C | na | 19946 | -0.013 | -0.1416 | No |
| 119 | PLD2 | na | 20094 | -0.014 | -0.1448 | No |
| 120 | RAB11A | na | 20153 | -0.014 | -0.1454 | No |
| 121 | EHD4 | na | 20372 | -0.016 | -0.1505 | No |
| 122 | SH3GLB1 | na | 20466 | -0.017 | -0.1519 | No |
| 123 | ERBB3 | na | 20473 | -0.017 | -0.1508 | No |
| 124 | SH3GL3 | na | 20477 | -0.017 | -0.1497 | No |
| 125 | WWP1 | na | 20539 | -0.018 | -0.1501 | No |
| 126 | HLA-A | na | 20544 | -0.018 | -0.1489 | No |
| 127 | DNM1L | na | 21040 | -0.022 | -0.1616 | No |
| 128 | STAM | na | 21098 | -0.023 | -0.1615 | No |
| 129 | HGS | na | 21308 | -0.025 | -0.1657 | No |
| 130 | CHMP4C | na | 21589 | -0.028 | -0.1718 | No |
| 131 | EGFR | na | 22041 | -0.033 | -0.1824 | No |
| 132 | RAB11FIP2 | na | 22148 | -0.034 | -0.1830 | No |
| 133 | AP2B1 | na | 22449 | -0.037 | -0.1890 | No |
| 134 | ADRB1 | na | 22618 | -0.039 | -0.1909 | No |
| 135 | CDC42 | na | 22626 | -0.039 | -0.1883 | No |
| 136 | VPS37A | na | 22782 | -0.041 | -0.1897 | No |
| 137 | CHMP1B | na | 22943 | -0.042 | -0.1912 | No |
| 138 | NEDD4 | na | 22944 | -0.042 | -0.1881 | No |
| 139 | DNM1 | na | 23055 | -0.044 | -0.1880 | No |
| 140 | IQSEC1 | na | 23151 | -0.045 | -0.1874 | No |
| 141 | GRK5 | na | 23415 | -0.048 | -0.1915 | No |
| 142 | AP2A1 | na | 23676 | -0.052 | -0.1951 | No |
| 143 | CLTB | na | 23684 | -0.052 | -0.1915 | No |
| 144 | VPS37D | na | 23700 | -0.052 | -0.1880 | No |
| 145 | HLA-B | na | 23866 | -0.054 | -0.1888 | No |
| 146 | EHD1 | na | 23993 | -0.056 | -0.1883 | No |
| 147 | RAB5B | na | 24209 | -0.059 | -0.1901 | No |
| 148 | GIT2 | na | 24307 | -0.060 | -0.1884 | No |
| 149 | RAB11FIP5 | na | 24384 | -0.062 | -0.1860 | No |
| 150 | VPS45 | na | 24840 | -0.068 | -0.1941 | No |
| 151 | IQSEC3 | na | 25067 | -0.072 | -0.1954 | No |
| 152 | IL2RA | na | 25190 | -0.073 | -0.1934 | No |
| 153 | SMAP2 | na | 25253 | -0.074 | -0.1897 | No |
| 154 | FGFR2 | na | 25276 | -0.075 | -0.1848 | No |
| 155 | ARFGAP3 | na | 25903 | -0.086 | -0.1965 | No |
| 156 | SMURF1 | na | 26141 | -0.090 | -0.1967 | No |
| 157 | RNF41 | na | 26307 | -0.094 | -0.1945 | No |
| 158 | HSPA6 | na | 26391 | -0.095 | -0.1898 | No |
| 159 | EHD2 | na | 26619 | -0.100 | -0.1890 | No |
| 160 | ASAP2 | na | 27677 | -0.123 | -0.2104 | No |
| 161 | CSF1R | na | 27843 | -0.127 | -0.2058 | No |
| 162 | DNM3 | na | 28124 | -0.134 | -0.2039 | No |
| 163 | SRC | na | 28777 | -0.151 | -0.2116 | No |
| 164 | SMURF2 | na | 29180 | -0.162 | -0.2112 | No |
| 165 | MET | na | 29439 | -0.170 | -0.2060 | No |
| 166 | EHD3 | na | 29852 | -0.184 | -0.2043 | No |
| 167 | GRK4 | na | 30031 | -0.189 | -0.1954 | No |
| 168 | ADRB3 | na | 30873 | -0.216 | -0.2037 | No |
| 169 | PIP5K1A | na | 32090 | -0.268 | -0.2189 | Yes |
| 170 | GRK1 | na | 32469 | -0.291 | -0.2082 | Yes |
| 171 | PARD6B | na | 32493 | -0.293 | -0.1872 | Yes |
| 172 | DNAJC6 | na | 32956 | -0.326 | -0.1764 | Yes |
| 173 | CXCR4 | na | 33022 | -0.330 | -0.1538 | Yes |
| 174 | SH3KBP1 | na | 33148 | -0.341 | -0.1321 | Yes |
| 175 | CXCR1 | na | 33350 | -0.357 | -0.1114 | Yes |
| 176 | GRK7 | na | 33698 | -0.390 | -0.0925 | Yes |
| 177 | CLTCL1 | na | 33772 | -0.401 | -0.0648 | Yes |
| 178 | ASAP1 | na | 34095 | -0.442 | -0.0413 | Yes |
| 179 | PIP5K1B | na | 34702 | -0.803 | 0.0008 | Yes |
Table: GSEA details [plain text format]

  

Fig 2: KEGG\_ENDOCYTOSIS      
 Blue-Pink O' Gram in the Space of the Analyzed GeneSet

  

Fig 3: KEGG\_ENDOCYTOSIS: Random ES distribution      
 Gene set null distribution of ES for **KEGG\_ENDOCYTOSIS**
